# Uncoupling of EGFR–RAS signaling and nuclear localization of YBX1 in colorectal cancer

**DOI:** 10.1038/oncsis.2015.51

**Published:** 2016-01-18

**Authors:** F Roßner, C Gieseler, M Morkel, H-D Royer, M Rivera, H Bläker, M Dietel, R Schäfer, C Sers

**Affiliations:** 1 Institute of Pathology, Charité Universitätsmedizin Berlin, Berlin, Germany; 2 DKTK, German Consortium for Translational Cancer Research, Partner site Berlin and DKFZ, German Cancer Research Center, Heidelberg, Germany; 3 Institute of Human Genetics and Anthropology, Heinrich Heine University Düsseldorf, Düsseldorf, Germany; 4 EPO, Experimental Pharmacology & Oncology Berlin-Buch GmbH, Berlin, Germany

## Abstract

The transcription factor YBX1 can act as a mediator of signals transmitted via the EGFR–RAS–MAPK axis. YBX1 expression has been associated with tumor progression and prognosis in multiple types of cancer. Immunohistochemical studies have revealed dependency between YBX1 expression and individual EGFR family members. We analyzed YBX1 and EGFR family proteins in a colorectal cancer (CRC) cohort and provide functional analyses of YBX1 in the context of EGFR–RAS–MAPK signaling. Immunohistochemistry for YBX1 and EGFR family receptors with two antibodies for YBX1 and EGFR were performed and related to clinicopathological data. We employed Caco2 cells expressing an inducible *KRASV12* gene to determine effects on localization and levels of YBX1. Mouse xenografts of Caco2-*KRASV12* cells were used to determine YBX1 dynamics in a tissue context. The two different antibodies against YBX1 showed discordant immunohistochemical stainings in cell culture and clinical specimens. Expression of YBX1 and EGFR family members were not correlated in CRC. Analysis of Caco2 xenografts displayed again heterogeneity of YBX1 staining with both antibodies. Our results suggest that YBX1 is controlled via complex regulatory mechanisms involving tumor stroma interaction and signal transduction processes. Our study highlights that YBX1 antibodies have different specificities, advocating their use in a combined manner.

## Introduction

Y-Box-binding protein 1 (YBX1) is the most prominent member of the Y-Box-binding protein family, comprising of transcription factors binding to DNA sequences called Y-Boxes.^1, 2, 3^ YBX1 has been associated with multiple cancer-related processes such as DNA-repair, extracellular stress response,^4, 5, 6, 7^ transcriptional^4, 8, 9, 10^ and translational control^8, 10, 11, 12^ as well as cell proliferation.^3, 13^ YBX1 was suggested to be a prognostic clinical biomarker in different cancer types and correlated with poor prognosis in breast cancer,^14, 15^ lung cancer,^16, 17^ multiple myeloma,^18^ osteosarcoma,^19^ synovial sarcoma,^20^ prostate cancer^21^ and in ovarian cancer.^22^ Recently, Woolley *et al.*,^23^ challenged the relevance of various prognostic data due to the fact that different YBX1 antibodies recognize specific but distinct epitopes and thereby provide incompatible information on YBX1 expression, nuclear or cytoplasmic localization.

YBX1 has also been singled out as an experimental therapeutic target. SiRNA-dependent knockdown unraveled a functional role of YBX1 in tumor invasion, proliferation and apoptosis.^18, 24, 25^ YBX1 is involved in chemotherapy response.^18, 19, 26^ Intracellular localization is essential for YBX1 function and a complex regulation underlies the translocation of YBX1 between cytoplasm and nucleus. Bargou *et al.*^27^ reported that nuclear localization of YBX1 is associated with drug resistance in human breast cancer. In lung cancer, nuclear localization of YBX1 is correlated with EGFR and LRP (lung resistance protein) expression.^28^ In colorectal cancer (CRC), YBX1 has been identified as a prominent mediator of malignant properties downstream of the EGFR–RAS–MAPK signaling cascade.^29^ NFY/YBX1-binding sites were overrepresented among RAS/MAPK target genes, YBX1 binding was enhanced on a group of RAS/MAPK target genes in *KRAS*-transformed CRC cells and nuclear YBX1 staining was detected in lung metastasis of CRC. A subgroup of genes targeted via YBX1 in CRC had been identified earlier as YBX1 target genes in breast cancer.^30^

Several levels of interaction exist between YBX1 and receptor tyrosine kinases of the EGFR family, consisting of EGFR (also known as ERBB1 and HER1) ERBB2 (also known as HER2), ERBB3 (HER3) and ERBB4 (HER4). In breast carcinoma cells, but not in CRC cells, YBX1 was found to bind to the promoter and act as a transcriptional activator of the *EGFR* gene.^31^ YBX1 mediated resistance to anti-ERBB2 therapy via a complex, RSK-dependent mechanism^32^ and prevents apoptosis in ERBB2-overexpressing breast cancer cells.^33^ In contrast to the well-known link between YBX1 and EGFR in breast or lung cancer, there is little knowledge about the interaction of YBX1 and the EGFR family in CRC.

The aim of this study is to examine a potential prognostic correlation between YBX1 and/or EGFR family expression in a large colon carcinoma cohort. We applied two antibodies against different epitopes of the YBX1 protein (YBX1n^27^ and YBX1c^3^) and examined the staining patterns. We also investigated YBX1 expression and its dependency on RAS signaling in *KRAS*-inducible transgenic colon cancer cell lines and mice, to provide a rationale for the observed sub-cellular localization of YBX1 in CRC tissue.

## Results

### Expression of YBX1 and EGFR family members is independent in CRC

We performed immunohistochemical staining for YBX1 in a cohort of 423 CRC samples using 2 different YBX1 antibodies directed against the C-terminal (YBX1c) and the N-terminal domain (YBX1n).

**Figure 1:**
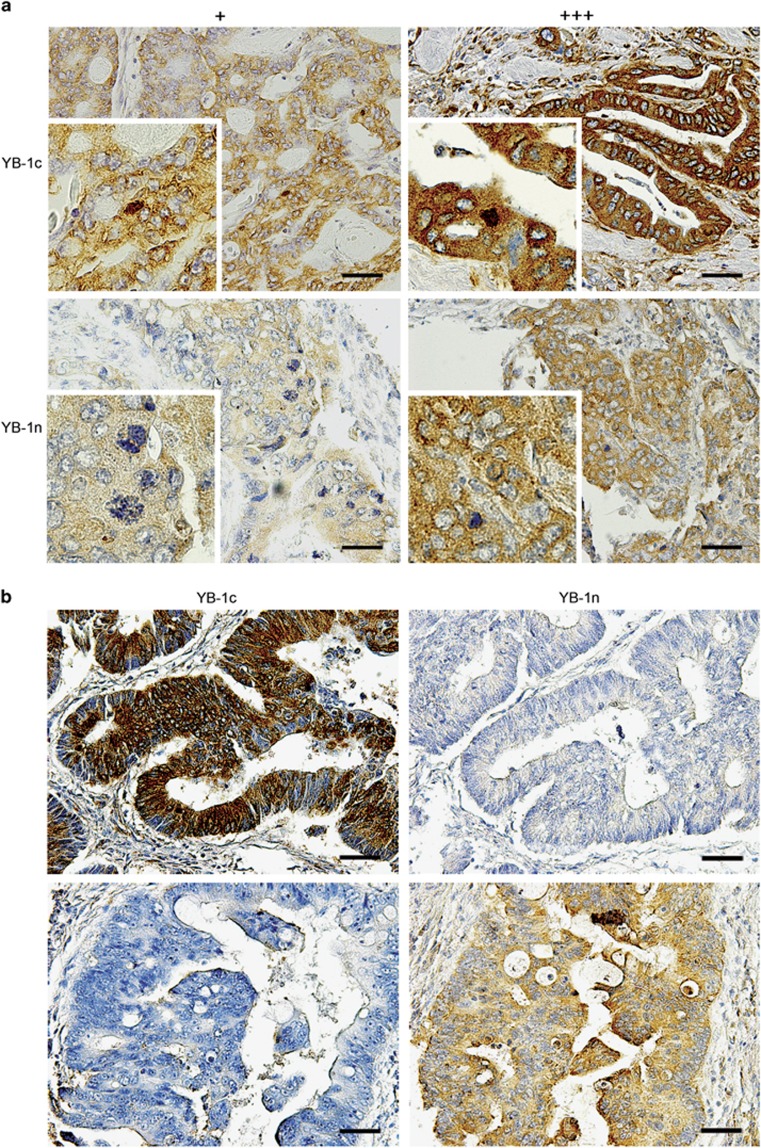
(**a**) Immunohistochemical staining of YBX1c (upper part) and YBX1n (lower part) with low (+) and high (+++) expression profiles. Magnification: × 200. Insets: nuclear staining, × 400 magnification. (**b**) Immunohistochemical staining of YBX1c and YBX1n showing the same tumor sample with marked differences in staining. Scale bar: 100 μm.

Assessing YBX1c antibody, we found weak cytoplasmic staining in 169 cases (39.9%), moderate staining in 76 cases (18.0%) and strong staining in 82 cases (19.4%). Ninety-six cases were negative (22.7%). We observed focal nuclear staining in six cases (1.4% Figure 1a upper panel). With YBX1n antibody, we observed weak cytoplasmic staining in 234 cases (55.3%), moderate cytoplasmic staining in 42 cases (9.9%) and strong cytoplasmic staining in 17 cases (4.0%). One hundred thirty cases (30.8%) were negative. Focal nuclear staining by YBX1n was observed in four cases (1.0% Figure 1a lower panel). These data indicate that antigen detection is stronger using the YBX1c antibody. In full section tumor tissue slides, we found a homogeneous staining result throughout the tumor. We did not detect differences in staining at the invasion front (Supplementary Figure S8). In total, the 2 YBX1 antibodies, despite being directed against the same protein, displayed discordant staining in 250 cases (Figure 1b). Sixty-one cases showed a low-expression pattern and 32 cases showed a high-expression pattern detectable with YBX1c, whereas YBX1n did not detect the antigen in these cases. Fifty-two specimens showed a low-expression pattern and seven showed a high-expression pattern by staining with YBX1n, whereas YBX1c was negative in these cases. Nuclear YBX1 protein was equally detected by YBX1c and YBX1n in three specimens (30%). In one case, YBX1n staining showed a focal nuclear pattern, whereas YBX1c staining did not detect nuclear YBX1.

**Figure 2:**
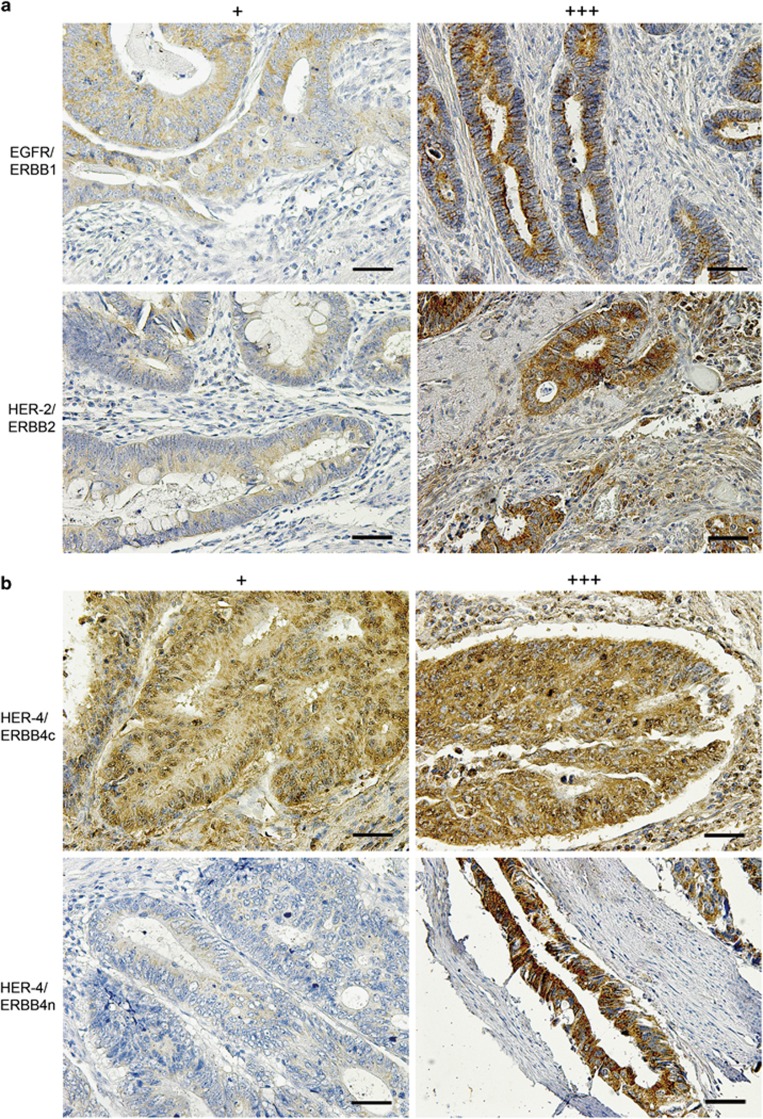
(**a**) Immunohistochemical staining of HER1/ERBB1, HER2/ERBB2 and with low (+) and high (+++) expression. (**b**) Immunohistochemical staining of c-terminal and n-terminal HER4/ERBB4 with low (+) and high (+++) expression. Magnification: × 200. Scale bar: 100 μm.

The EGFR receptor revealed a diffusely distributed cytoplasmic granular pattern (Figure 2a). Two hundred thirty six cases (55.8%) were weakly positive, 61 cases (14.4%) stained moderately and a strong cytoplasmic staining was observed in 19 cases (4.5%), while 107 cases (25.3%) were negative. The ERBB2 receptor displayed a regular distributed cytoplasmic staining, with 258 cases (61.0%) showing weak cytoplasmic staining, 63 cases (14.9%) stained moderately and 15 cases (3.5%) showed a strong cytoplasmic staining (Figure 2a). No staining was observed in 87 cases (20.6%). C-terminal ERBB4 antibody showed a weak cytoplasmic signal in 219 cases (51.8%), moderate staining in 53 cases (12.5%) and strong staining in 3 cases (0.7% Figure 2b). No staining was observed in 148 cases (35.0%). The N-terminal ERBB4 antibody showed weak cytoplasmic staining in 144 cases (34.0%), moderate cytoplasmic staining in 34 cases (8.0%) and strong cytoplasmic staining in 18 cases (4.3%). No staining was observed in 227 cases (53.7% Figure 2b). The 2 ERBB4 antibodies displayed discordant staining in 266 cases (Supplementary Figure S2). One hundred fourteen specimens showed a low-expression pattern and 28 cases showed a high-expression pattern detectable by ERBB4c staining, whereas the ERBB4n antibody showed no staining in these cases. ERBB4n staining showed low antigen expression patterns in 56 cases and high-expression patterns in 7 specimens, whereas ERBB4c showed no staining in these cases. As expected, nuclei were never stained with any antibody directed against EGFR family members. Data for ERBB3 in CRC will be presented elsewhere (manuscript in preparation). Full section tumor tissue slides showed only faint and inconspicuous intra-tumor staining differences. ERBB4 antibodies showed more differences in 10% (ERBB4n) and 30% (ERBB4c) of the tumor tissue (Supplementary Figures S3 and S4).

We correlated the staining patterns for YBX1 and the EGFR family members using a *χ*^2^-test. Importantly, we could not detect a significant correlation between YBX1 expression and patterns obtained for any of the ERBB proteins, also irrespective of the intracellular localization of YBX1 (Table 1). Comprehensive assessment of discrepancy levels between the antibodies confirmed previous findings (Supplementary Figure S5). The vast majority of tumors showed only 1–5 of 12 score points expression difference between the antibodies. YBX1n and EGFR displayed the highest number of cases with concordant staining (*n*=115/27, 2%), YBX1c and EGFR had the fewest number of cases with concordant staining (*n*=49/11, 6%).

### Statistical analyses of clinical parameters and survival analysis

*χ*^2^-tests indicate that YBX1n positivity, but not YBX1c positivity, obtained in primary CRC reached a borderline significant negative correlation with the propensity to metastasize (Pearson—*χ*^2^: 4.09; *P*=0.043), that is, a lower number of metastasized tumors were scored YBX1n positive than expected (residual value: −6.4; Table 2A). Patients with YBX1n-positive tumors (independent of the localization) exhibited increased survival compared with patients with YBX1n-negative tumors (*P*=0.016; Supplementary Figure S6C). A tendency (*P*=0.45) is also visible on splitting tumors into low- and high-YBX1 expression subgroups (Supplementary Figure S6D). YBX1c staining did not show significant association with clinical parameters and Kaplan–Meier survival analysis showed no advantage in survival for YBX1c-negative tumors (*P*=0.977), nor was there a significant difference between high- and low-expression subgroups (*P*=0.922; Supplementary Figure S6A and B).

ERBB2 expression is statistically significantly correlated with tumor localization (*P*=0.005), with a higher number of ERBB2-positive cases in the left colon. Further correlations between EGFR family members (ERBB1; ERBB2; ERBB4) with clinicopathologic data were not significant (Tables 2B and C). Multilinear regression analysis showed that expression patterns obtained with antibodies against YBX1c, YBX1n and the EGFR family proteins are not correlated with clinicopathologic data (Supplementary table S1).

### EGFR/RAS signaling can modulate YBX1 localization *in vivo* in CRC cells and intestinal tissues

The immunohistochemical investigation revealed a low number of specimens with nuclear YBX1, although we have found nuclear YBX1 in a limited set of pulmonary metastases of CRC before.^27^ We therefore investigated functional mechanisms contributing to differential YBX1 localization.

**Figure 3:**
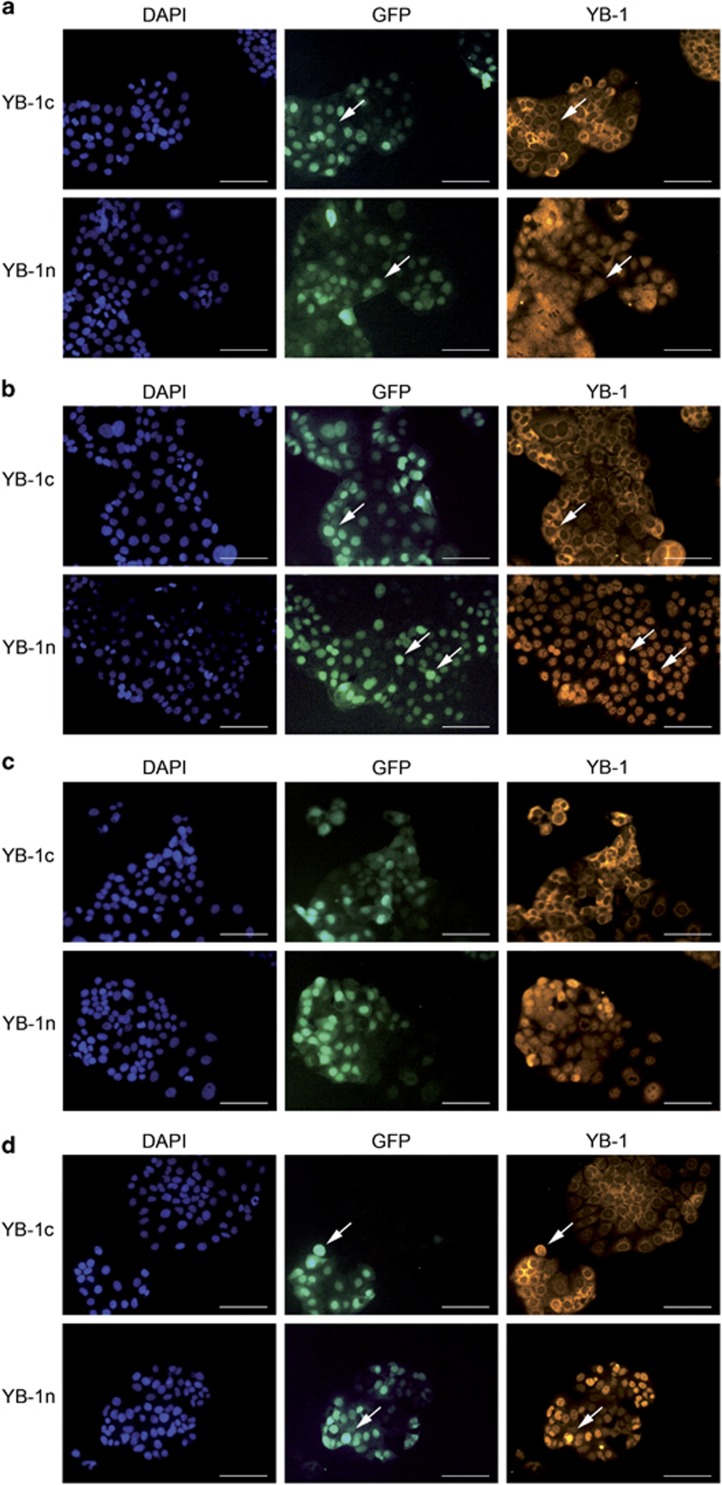
Immunofluorescence of cultured Caco2 cells showing staining of YBX1n and YBX1c after 24 h (**a**), 48 h (**b**), 72 h (**c**) and 96 h (**d**) of doxycycline (2 μg/ml) treatment. DAPI: nuclear stain, GFP: RAS expression, YB-1: cytoplasmic YBX1c and nuclear YBX1n. Arrows indicating nuclear YBX1n and cytoplasmatic YBX1c stainings (**a**) and enhancement after RAS induction (**b**,**d**). Scale bar: 100 μm.

We used Caco2 CRC cells harboring an inducible *KRAS*^*V12*^ oncogene/green fluorescent protein (GFP) transgene and tested YBX1 expression via immunofluorescence. In non-induced cells, we observed strictly distinct staining patterns using YBX1c and YBX1n antibodies. The YBX1c antibody displayed a cytoplasmic perinuclear staining, while the YBX1n antibody stained the protein in the nucleus (Figure 3a). Following RAS induction, the YBX1n-positive signals first accumulated in a few nuclei (Figure 3b) and after 96 h a strong and condensed nuclear YBX1n signal was detected, whereas the YBX1c signal remained perinuclear. Both, YBX1c and YBX1n staining intensity increased after *KRAS*^*V12*^ induction in a subset of cells. Other cells in direct neighborhood did not show increased staining despite efficient RAS activation (as judged by GFP fluorescence linked to KRAS).

**Figure 4:**
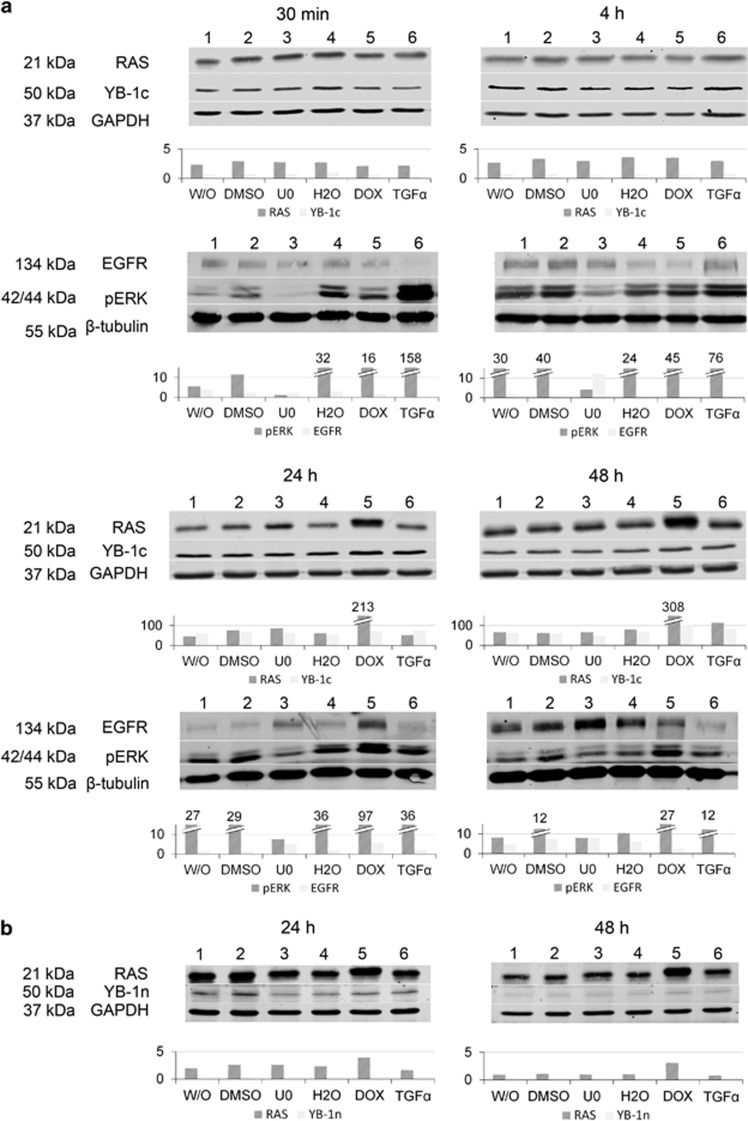
(**a**) Expression of RAS, pERK and YBX1c in Caco2 cells following KRAS induction for 30 min, 4 , 24  and 48 h. RAS and phospho-ERK1/2 are induced after 24 and 48 h following doxycycline treatment. Phospho-ERK increases strongly after 30 min and 4 h following TGFα application; EGFR is induced 48 h after U0126 treatment. W/O (1), DMSO (2), U0126 (3), aqua bidest (4), doxycycline (5), TGFα (6). (**b**) RAS is upregulated after 24 h and 48 h of doxycycline treatment; again no change of YBX1n. W/O (1), DMSO (2), U0126 (3), aqua bidest (4), doxycycline (5), TGFα (6).

We also tested the localization of YBX1 in the intestine of transgenic mice harboring an inducible *KRAS*^*V12*^ transgene. In the non-induced intestine, both antibodies showed robust YBX1 protein expression, which was cytoplasmic in the villus but cytoplasmic and nuclear in the crypt compartment. In contrast, 4 days following induction of the *KRAS*^*V12*^ transgene both antibodies displayed combined cytoplasmic and nuclear signals throughout the crypt–villus axis (Supplementary Figure S7). To test a potential increase in YBX1 total protein levels following RAS or EGFR activation, we performed western blot analysis of Caco2 cells after *KRAS*^*V12*^ induction and after addition of the EGFR ligand TGFα. We also tested the impact of MEK inhibition. Transgenic RAS expression became visible 24 h following doxycycline-induction and was most prominent after 48 h (Figure 4). Concomitant activation of MAPK signaling was evident via increased pERK levels, however, there was no change in YBX1c-positive or EGFR-positive cells. Likewise, treatment of the cells with TGFα-induced pERK levels (Figure 4a, 30 min; 4 h), however, there was no effect on YBX1c staining. Similar results were obtained using the YBX1n antibody, albeit with weaker intensity due to a lower sensitivity of the antibody on western blots (Figure 4b). These results indicate that nuclear localization of YBX1 is detectable prior to, but accentuated, following KRAS activation and are consistent with a preferential detection of nuclear YBX1 using the YBX1n antibody. The total level of YBX1 protein, however, is not increased following activation of the EGFR–RAS–MAPK axis.

**Figure 5:**
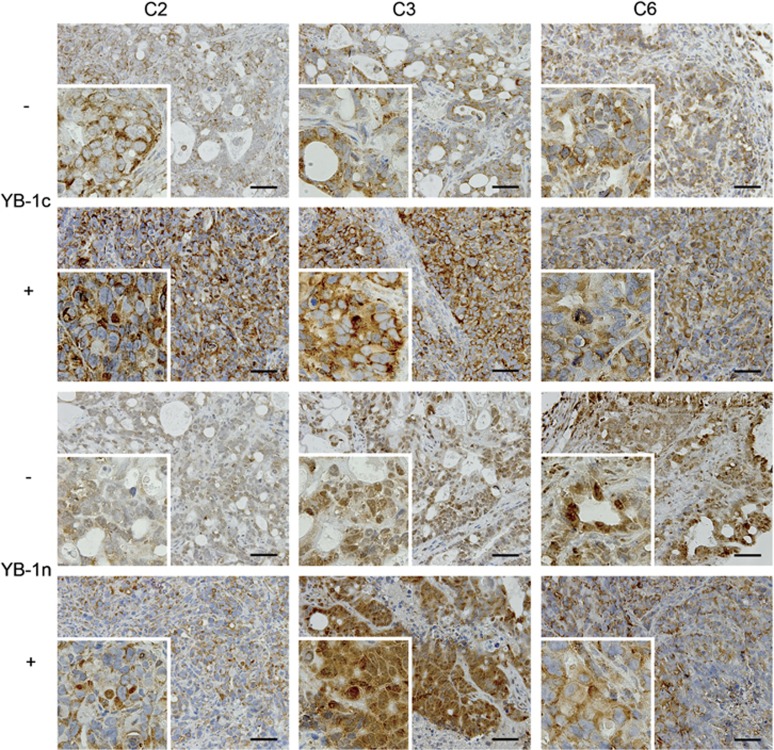
Immunohistochemical staining using YBX1n and YBX1c antibodies in mouse xenografts of Caco2 cells prior to (−) or following KRAS oncogene induction (+). Three independent tumors, C2, C3 and C6 were stained. Scale bar: 100 μm.

The rather uniform localization of YBX1 in the nucleus of KRAS-induced Caco2 cells *in vitro* using the YBX1n antibody was in contrast to our previous finding of low numbers of CRC specimens, in which nuclear YBX1 was detectable. We therefore asked whether the tumor microenvironment could modulate YBX1 expression. We determined YBX1 expression and localization in xenografts from *KRAS*^*V12*^-inducible Caco2 in the absence and presence of doxycycline, yielding *KRAS*^*V12*^-negative and -positive tumors. We found no significant difference in *YBX1* and *EGFR* mRNA levels between *KRAS*^*V12*^-negative and *KRAS*^*V12*^-induced mouse xenograft samples (data not shown). At the protein level, we detected a moderate YBX1 staining in non-induced xenograft tumors. The YBX1c staining pattern was largely cytoplasmic in non-induced xenografts. Most interestingly, there was a clear nuclear staining detectable using the YBX1n antibody (such as tumor C3, Figure 5). Staining was stronger in xenografts derived after *KRAS*^*V12*^ induction as revealed with the two antibodies (Figure 5). YBX1c staining remained mainly cytoplasmic, however, occasional focal nuclear staining was visible (Figure 5, upper panel, inserts C2 and C3). YBX1n showed a more variable staining pattern within and between the individual mouse samples. The heterogeneity of YBX1 protein expression and spatial distribution observed with both antibodies was significantly higher in Caco2 xenografts as compared with the cell cultures. This indicates that *in vivo*, both YBX1 protein levels and intracellular localization are most likely controlled via additional stromal effects, beyond activation of KRAS.

## Discussion

With this study we aimed to scrutinize the role of YBX1 as a prognostic marker in a large cohort of CRCs, and to determine any functional connection between EGFR, RAS–MAPK signaling and YBX1 expression. We observed striking differences between YBX1 patterns detected either with YBX1n and YBX1c antibody (Figure 1b). It was found previously that YBX1 staining can depend on the choice of antibody. Woolley *et al.*^23^ assigned these variations to different affinities to the target and affected by phosphorylation-induced conformational changes of the protein.^34, 35, 36, 37^ Yet, this theory was disproved by testing phosphorylated YBX1 in complexes immunoprecipitated with either antibodies.^23^

We saw that differences in antigen staining patterns between YBX1c and YBX1n antibodies extended beyond clinical specimens, as they were also obvious in immunofluorescence analyses of Caco2 cells. The molecular events controlling YBX1 shuttling and sub-cellular localization have been delineated only incompletely until now. Raffetseder *et al.*^38^ described that YBX1 nuclear localization is actively mediated via the splicing factor SRp30c/SRSF9. Furthermore, Stein *et al.*^39^ described a heat-shock-induced, rapid nuclear accumulation of YBX1-inducing *MDR1* transcription, which declined again within 4 h following heat shock. Both examples indicate that cytoplasmic-nuclear shuttling of YBX1 is a dynamic process. Woolley *et al.*^23^ demonstrated by immunoprecipitation and gel electrophoresis that the antibodies against YBX1 recognize the phosphorylated protein presumably in different complexes. The authors concluded that one of the YBX1 epitopes is likely to be masked within one of the complexes in the nucleus under conditions of immunohistochemical staining. Our analysis revealed several cases in which the YBX1c and YBX1n reacted discordantly on the same tumor under identical processing conditions (Figure 1b). This observation could indicate a tissue processing effect affecting the two epitopes in a different way and rendering either the nuclear or the cytoplasmic YBX1 inaccessible. Thus, a negative YBX1 staining with only one of the two antibodies does not indicate that YBX1 is not expressed or present at very low level, but rather suggests an intracellular molecular complex formation, which cannot be disregarded as an artifact of tissue processing. Elucidation of the functional relevance of these complexes will require detailed biochemical analysis.

In our human tumor cohort, we observed low numbers with focal nuclear staining of YBX1c and YBX1n. Regardless of the different results obtained with the antibodies employed, we conclude that nuclear localization of YBX1 is rare in primary CRC. Other reports also describe low percentages of nuclear YBX1 using immunohistochemistry in breast cancer cohorts^23, 40^ The overall (cytoplasmic plus nuclear) presence of YBX1 seemed sufficient for establishing prognostic relevance in several tumor entities.^41, 42, 43^ A separate assessment of nuclear and cytoplasmic staining might not be necessary. With our analysis, we were not able to define a significant prognostic impact for YBX1 staining in primary colon cancer. The current results qualify our previous study, suggesting a trend (*P*=0.076) towards reduced survival of patients with strong YBX1 cytoplasmic staining in an independent set of 118 CRC samples and a significant correlation between high nuclear staining and reduced survival in pulmonary metastases (*P*=0.005).^29^ We suggest that correlations between nuclear YBX1 and patient outcome currently cannot be established in primary CRC possibly owing to the fact that YBX1 translocation to the nucleus is transient and considerably rare in non-metastatic colonic tissue specimens. Therefore, cytoplasmic YBX1 staining likewise does not represent a robust prognostic marker. Our results on tissue micro arrays (TMAs) are strengthened further, since staining of full section tumor tissues did not reveal significant expression heterogeneity, whether it is intratumoral or at the invasion front, for both YBX1 antibodies (Supplementary Figures S3 and S8).

Immunohistochemical analysis of EGFR, ERBB2 and ERBB4 receptors revealed distinct patterns for the different receptors (Figure 2). Full section tumor tissue showed only faint and defined intra-tumor discrepancies for ERBB4 in low percentages of tumor tissue, but eventually these are more likely fixation artefacts than true expression differences (Supplementary Figure S4). In contrast to findings in NSCLC and breast cancer, we could not define a statistically significant correlation between expression of YBX1 and EGFR family receptors in our large CRC cohort.^28, 44, 45^ Specifically, neither a correlation between YBX1c/n with EGFR staining nor an impact onto EGFR in Caco2 cells was visible. While this indicates that YBX1 and EGFR are uncoupled in CRC, further functional testing is required to exclude this relationship, for example, at the level of receptor phosphorylation. While Fujii *et al.*^46^ described ERBB2 dependency on nuclear YBX1 in breast cancer, another study stated that ERBB2 is not dependent on YBX1 expression in breast cancer.^41^ Similar to the latter study, no correlation could be determined between YBX1 and ERBB2 in CRC in our cohort. With some borderline significant exceptions, we did not find compelling links between YBX1 and the EGFR family with clinicopathological parameters in CRC.

Further investigations of the mechanisms of YBX1 expression and localization *in vitro* and *in vivo* revealed that oncogenic KRAS induction can lead to a condensed expression of YBX1 in Caco2 cells *in vitro* and to an increased nuclear shuttling in intestinal epithelial cells in the mouse *in vivo*. These results provided compelling evidence that YBX1 activity is influenced and functionally tied to a strongly activated RAS/MEK/ERK signaling pathway in certain experimental settings. We found highly variable patterns of YBX1 protein localization in mouse xenografts derived from the same KRAS-inducible Caco2 cells used for *in vitro* experiments, whereas gene expression levels of *YBX1* and *EGFR* seemed not to be dependent on RAS signaling. These important results suggest that YBX1 expression and localization is controlled by a complex and as yet underappreciated regulatory network, and may strongly depend on the microenvironment of tumor cells. It is important to notice that prognostic statements and conclusions regarding YBX1 cannot be easily made without appreciation of contextual molecular data, the histological setting and the antibodies applied as diagnostic tools.

## Materials and methods

### Patient cohort

Our study was carried out on 423 cases of CRC paraffin-embedded tissue specimens (years 1995–2012). All patients provided informed consent at the Charité University Hospital for non-commercial and fully anonymous tissue use for research purposes. Two hundred and thirty five patients (55.6%) were male, 188 (44.4%) were female. Tumors were derived from different parts of the colon. Three hundred and three cases had a Grade 2 status (71.6%). 249 cases (58.9%) infiltrated the subserosa (pT3 stadium). In 40 cases (9.5%), the tumor was metastasized. KRAS mutation status was available for 380 samples. About 53 tumors were KRAS mutated (13.9%). BRAF mutation status was available for 58 samples and 5 tumors were BRAF mutated (8.6%) (Table 3).

### Immunohistochemistry

We manufactured TMAs from the study cohort tissue. To evaluate intra-tumor expression discrepancies and to thoroughly exclude a sampling bias, we also evaluated representative full section tumor tissues with high (score: 12) expression profile. TMAs were manufactured as described.^47^ Three representative regions with vital tumor tissue within the donor block were chosen. TMAs, Caco2 cell line xenografts and mouse intestine sections were cut into 2-μm slices and placed on glass slides. Excessive paraffin was melted off in a microwave at 70 °C. Tissue slides were incubated three times in 100% Xylol and descending ethanol series of 100, 96, 90 and 80% and twice in 70% ethanol each followed by aqua bidest washing. Antibody-dependent citrate- (1,97 mM, pH 6,0; Merck Millipore, Merck KGaA, Darmstadt, Germany) or EDTA-buffered (1,71 mM, pH 7,8; Sigma-Aldrich Corporation, St Louis, MO, USA) antigen-retrieval and cooling was performed according to the manufacturer's product guide followed by two aqua bidest/TBS-washing steps. Blocking solution was coated on HybriSlips (Sigma-Aldrich Corporation) and the slides were incubated. Blocking solution was removed with aqua bidest and TBS-washing solution and the primary antibody was applied overnight at 4 °C. The YBX1 antibodies were previously described.^3, 27^ The appropriate dilutions for YBX1 antibodies^27^ against the C-terminal epitope (*YBX1c*) and the N-terminal epitope (*YBX1n*) were tested in distinct experiments on tumor tissue and normal mucosa. EGFR (rabbit monoclonal EGF Receptor D38B1,#4267; dilution 1:100; Cell Signaling Technology, Cambridge, UK), HER2/ERRB2 (rabbit polyclonal HER2/ERBB2 #2242; dilution 1:50; Cell Signaling Technology), HER3/ERBB3 (rabbit polyclonal ERBB3 Antibody LS-C90418; dilution 1:100; LifeSpan Biosciences, Inc., Seattle, WA, USA), c-terminal HER4 (rabbit polyclonal ERBB4 LS-C97506; dilution 1:100; LifeSpan Biosciences, Inc.) and n-terminal HER4/ERRB4 (polyclonal rabbit ERBB4 N-term, #AP7631a; dilution 1:50; Abgent, San Diego, CA, USA) and RFP (anti-RFP, #600-401-379; dilution 1:200, Rockland, Gilbertsville, PA, USA) were applied according to the manufacturer's guide. For the staining procedure, we used the Dako REAL-kit (Dako Denmark A/S, Glostrup, Denmark). Anti-RFP was processed with ImmPRESS secondary antibody and NovaRED substrate kits (Vector Labs, Burlingame, CA, USA). IHC Slides were washed in TBS and the secondary antibody was applied. The slides were washed again in TBS and the HRP-link was applied. At last, the chromogen substrate diluted in buffer solution was incubated on the slides and a proper staining degree was determined with light microscopy. Staining reaction was stopped within a water bath. Hematoxylin core staining solution was applied, followed by alcohol fixation and cover-slip. For negative controls the primary antibody was omitted (Supplementary Figure S1). Pictures were taken with an Olympus BX53 light microscope linked to a DP25 camera (Olympus Corporation, Tokyo, Japan).

### Cell culture and immunofluorescence

We used a previously modified Caco2 cell line stably transfected with a doxycycline-inducible KRAS^G12V^,^48^ which allows for conditional *KRAS* expression. Cells were grown at 37 °C, 5% CO_2_ in Dulbecco's Modified Eagle's Medium (DMEM; Life Technologies, Thermo Fisher Scientific, Carlsbad, CA, USA) supplemented with 10% v/v FBS, Penicillin G (100U/ml) and Streptomycin (100 μg/ml; D10 medium) with Puromycin (5 μl/ml) and Blasticidin (5 μg/ml). Cells were plated at 1x10^4^ cells per chamber (24-h measurement) or 1.25 × 10^3^ cells per chamber (96-h measurement) in an eight-well chamber slide system (Lab-Tek II Chamber Slide, Thermo Fisher Scientific, Inc., Rochester, NY, USA). For protein extraction and western blotting, 2.5 × 10^4^ cells per well (24-h measurement) or 1.25x10^4^ cells per well (96-h measurement) were plated in 60 mm dishes. KRAS^G12V^ was induced with Doxycyclin at 2 μg/ml. DMSO was added as a control at 10 μM. The MEK inhibitor U0126 was added at 10 μM; TGFα was added at 20 ng/ml (30 min and 4 h) and 10 ng/ml (24 and 48 h). Prior to immunofluorescence, cells were fixed with 4% of formaldehyde at room temperature. Fixative was removed, cells were washed with 1x PBS and blocking solution containing 5% BSA was applied. Blocking solution was aspirated and YBX1c and YBX1n were applied in a 1:100 dilution and incubated overnight at 4 °C. The primary antibody was removed, the cells were washed with TBS three times and the secondary antibody (Red Fluorescent AlexaFluor 546 goat anti-rabbit IgG, Life Technologies, Thermo Fisher Scientific) was applied. After TBS rinsing the washing solution was removed. The chamber grid was carefully detached. The silicone gasket was removed with a thin-bladed spatula, the slides were coverslipped using a 2 μg/ml 1,4-Diazabicyclo[2.2.2]octane-based solution (460 mg, 18 mg Glycerin and 2 ml 0.2 m Tris solution at pH 8.0) and stored in a dark environment at 4 °C. Pictures of Immunofluorescence stains were taken using a Zeiss Axiovert 40 CFL microscope linked to an AxioCam MRc camera (Carl Zeiss AG, Oberkochen, Germany).

### Immunohistochemical evaluation

Immunohistochemical staining was evaluated independently by two pathologists (HB and FR). An immunoreactive score (IRS)^49^ was applied, which was calculated as a product of staining intensity and the percentage of stained tumor cells (0=no staining visible, 1=weak staining, 2=moderate staining, 3=strong staining) and percentage of positive cells (0=no cells, 1⩽10% positive, 2=10–50% positive, 3=50–80% positive, 4⩾80% positive). Tumor samples with a result between 0 and 6 (group1) or between 8 and 12 (group2) were assigned to subgroups with low-expression pattern^1^ and high-expression pattern.^2^

### Western blot

About 40 μg of whole cell lysates per lane were separated by SDS–PAGE at 100mA for 60 min. LI-COR blocking buffer (LI-COR Biosciences, Lincoln, NE, USA) and PBS solution was applied 1:1 for 60 min. We used Pan-RAS primary antibody (mouse monoclonal Anti-Pan-Ras, MABS195; dilution 1:500; Merck Millipore, Merck KGaA, Darmstadt, Germany) for detection of total RAS protein. YBX1c and YBX1n antibodies were applied at 1:1500. GAPDH served as a control (mouse monoclonal GAPDH, clone 6C5, AM4300; dilution 1:15000; Life Technologies, Thermo Fisher Scientific). EGF receptor (rabbit monoclonal EGF Receptor D38B1, #4267; dilution 1:1000) and phospho-MEK1/2 (rabbit polyclonal Phospho-MEK1/2 (Ser217/221), #9121; dilution 1:1000; Cell Signaling Technology) were used for western blot analysis. β-Tubulin (rabbit polyclonal β-Tubulin, #2146; dilution 1:1000; Cell Signaling Technology) served as a loading control in the latter western blot analyses. Anti-rabbit and anti-mouse antibodies coupled to Fluorescent AlexaFluor Dyes (Green Fluorescent Red Fluorescent AlexaFluor 488 goat anti-mouse IgG and Red Fluorescent AlexaFluor 546 goat anti-rabbit IgG, dilution 1:10000) were applied as secondary antibodies and detected with an infrared imaging system (Odyssey, LI-COR Biosciences, Lincoln, NE, USA). For semi-quantitative evaluation, YBX1 and EGFR values were normalized against GADPH and β-Tubulin levels.

### Mouse intestine samples and Caco2 cell-derived xenografts

Transgenic mice harboring oncogenes in the Gt(ROSA)26Sor locus were described previously.^50, 51^ For short-term KRASV12 induction *in vivo*, mice were provided doxycycline at 4 mg/ml in a 1% sucrose solution via the drinking water for 3–4 days. Induced and control mice were killed, and intestines (Jejunum/Ileum) were fixed for 12–24 h in a 4% formalin solution, before they were dehydrated and embedded in paraffin. For immunohistochemistry, 4 μm sections were used, as described above. Experiments were approved by Berlin authorities LAGeSo (G0185/09).

For xenografts, 3x10^6^ Caco2 cells stably transfected with a doxycycline-inducible KRAS^G12V^^48^ and suspended in a 1:1 mixture of matrigel and PBS were injected s.c. into NMRI nu/nu and NSG mice (6 mice per group). For transgene induction, 2 mg/ml doxycycline (in 5% sucrose) was administered ad libitum starting at day 1 after injection. Mice were observed for 35 days and maintained under sterile and controlled conditions (22 °C, *ca.* 50% relative humidity, 12 h light–dark cycle, autoclaved food and bedding, acidified drinking water). Tumor diameters were determined 2 × weekly by caliper measurements and tumor volume was determined with the formula TV=(width^2^ x length x 0.5); body weight was measured as a parameter for tolerability. Xenograft experiments were approved by Berlin authorities LAGeSo (A0452/08).

### Statistical analysis

Data sets were administrated with Microsoft Access 2003, statistical analysis was performed using the SPSS 21.0 software (SPSS, Chicago, IL, USA). Survival analyses were calculated with Kaplan–Meier functions and Log-rank test. Linear regression model and *χ*^2^-test were used to describe the correlation between expression data and clinicopathological data. A non-parametric correlation test (Spearman-Rho) was applied to describe the correlations between expression data. All tests were two-sided and *P*-values <0.05 were considered statistically significant.

## Figures and Tables

**Table 1:** Spearman's rank correlation showing YBX1 against HER receptor expression

	*HER1*	*HER2*	*HER4c*	*HER4n*
*YBX1c*	0.056	0.038	−0.007	0.085
Sig. (two-sided)	0.254	0.432	0.892	0.080
*N*	423	423	422	423
*YBX1n*	0.069	−0.016	0.003	−0.044
Sig. (two-sided)	0.154	0.743	0.950	0.372
* N*	423	423	422	423

**Table 2A:** Cross-tables of expression of YBX1c and YBX1n

*Parameter*	*Total (%)*	*YBX1c positive (%)*	*YBX1c negative (%)*	P-*value*	*YBX1n positive (%)*	*YBX1n negative (%)*	P-*value*
All cases	423 (100)	327 (77)	96 (23)		293 (69)	130 (31)	
							
*Age*							
>72	238 (56)	192 (81)	46 (19)	0.061	172 (72)	66 (28)	0.129
<72	185 (44)	135 (73)	50 (27)		121 (65)	64 (35)	

*Gender*							
Male	235 (56)	186 (79)	49 (21)	0.311	158 (67)	77 (33)	0.311
Female	188 (44)	141 (75)	47 (25)		135 (72)	53 (28)	

*pT status*							
pT1/pT2	98 (23)	77 (79)	21 (21)	0.697	71 (72)	27 (28)	0.48
pT3/pT4	313 (74)	240 (77)	73 (23)		215 (69)	98 (31)	

*Nodal status*							
N0	214 (51)	167 (78)	47 (22)	0.716	149 (70)	65 (30)	0.871
N1	209 (49)	160 (77)	49 (23)		144 (69)	65 (31)	

*Metastasis*							
M0	369 (87)	289 (78)	80 (22)	0.193	262 (71)	107 (29)	0.043
M1	54 (13)	38 (70)	16 (30)		31 (57)	23 (43)	

*Grade*							
G1	4 (1)	2 (50)	2 (50)	0.177	1 (25)	3 (75)	0.054
G2/G3	412 (98)	322 (78)	90 (22)		287 (70)	125 (30)	

*R status*							
R0	377 (89)	290 (77)	87 (23)	0.126	263 (70)	114 (30)	0.876
R1	22 (5)	20 (90)	2 (10)		15 (68)	7 (32)	

*Localization*							
Right colon	175 (41)	136 (78)	39 (22)	0.845	118 (67)	57 (33)	0.235
Left colon	225 (53)	173 (77)	52 (23)		164 (73)	61 (27)	

*KRAS status*							
WT	327 (77)	255 (78)	72 (22)	0.836	227 (69)	100 (31)	0.954
MUT	53 (13)	42 (79)	11 (21)		37 (70)	16 (30)	

**Table 2B:** Cross-tables of expression of HER1/ERBB1 and HER2/ERBB2

*Parameter*	*HER1 positive (%)*	*HER1 negative (%)*	P-*value*	*HER2 positive (%)*	*HER2 negative (%)*	P*-value*
All cases	306 (72)	107 (25)		336 (79)	87 (21)	
*Age*						
>72	175 (74)	63 (26)	0.528	184 (77)	54 (23)	0.221
<72	141 (76)	44 (24)		152 (82)	33 (18)	
						
*Gender*						
Male	182 (77)	53 (23)	0.147	191 (81)	44 (19)	0.294
Female	134 (71)	54 (29)		145 (77)	43 (23)	
						
*pT status*						
pT1/pT2	72 (73)	26 (27)	0.7	75 (77)	23 (23)	0.394
pT3/pT4	236 (75)	77 (25)		252 (81)	61 (19)	
						
*Nodal status*						
N0	164 (77)	50 (23)	0.355	170 (79)	44 (21)	0.997
N1	152 (73)	57 (27)		166 (79)	43 (21)	
						
*Metastasis*						
M0	278 (75)	91 (25)	0.433	294 (80)	75 (20)	0.747
M1	38 (70)	16 (30)		42 (78)	12 (22)	
						
*Grade*						
G1	3 (75)	1 (25)	0.974	4 (100)	0 (0)	0.305
G2/G3	306 (74)	106 (26)		326 (79)	86 (21)	
						
*R status*						
R0	280 (74)	97 (26)	0.429	301 (80)	76 (20)	0.771
R1	18 (82)	4 (18)		17 (77)	5 (23)	
						
*Localization*						
Right colon	126 (72)	49 (28)	0.123	127 (73)	48 (27)	0.005
Left colon	177 (79)	48 (21)		189 (84)	36 (16)	
						
*KRAS status*						
WT	241 (74)	86 (26)	0.079	261 (80)	66 (20)	0.924
MUT	45 (85)	6 (15)		42 (79)	11 (21)	

**Table 2C:** Cross-tables of c-terminal HER4/ERBB4 and n-terminal HER4/ERBB4

*Parameter*	*HER4c positive*	*HER4c negative*	P*-value*	*HER4n positive*	*HER4n negative*	P-*value*
All cases	276 (65)	147 (35)		196 (46)	227 (54)	
*Age*						
>72	154 (65)	84 (35)	0.79	102 (43)	136 (57)	0.104
<72	122 (66)	63 (34)		94 (51)	91 (49)	
						
*Gender*						
Male	149 (63)	86 (37)	0.373	114 (49)	121 (51)	0.316
Female	127 (68)	61 (32)		82 (44)	106 (56)	
						
*pT status*						
pT1/pT2	62 (63)	36 (37)	0.686	42 (43)	56 (57)	0.511
pT3/pT4	205 (66)	108 (34)		146 (47)	167 (53)	
						
*Nodal status*						
N0	140 (65)	74 (35)	0.94	92 (43)	122 (57)	0.163
N1	136 (65)	73 (35)		104 (50)	105 (50)	
						
*Metastasis*						
M0	242 (66)	127 (34)	0.706	165 (45)	204 (55)	0.081
M1	34 (63)	20 (37)		31 (57)	23 (43)	
						
*Grade*						
G1	3 (75)	1 (25)	0.678	1 (25)	3 (75)	0.383
G2/G3	268 (65)	144 (35)		193 (47)	219 (53)	
						
*R status*						
R0	245 (65)	132 (35)	0.458	174 (46)	203 (54)	0.443
R1	16 (73)	6 (27)		12 (55)	10 (45)	
						
*Localization*						
Right colon	117 (67)	58 (33)	0.615	85 (49)	90 (51)	0.277
Left colon	145 (64)	80 (36)		97 (43)	128 (57)	
						
*KRAS status*						
WT	212 (65)	115 (35)	0.479	154 (47)	173 (53)	0.449
MUT	37 (70)	16 (30)		22 (42)	31 (58)	

**Table 3:** Study cohort characteristics, *n*=423

	*Mean*	*s.d.*
*Age*		
	72.45	10.78
	*Frequency*	*Percentage*
		
*Sex*		
Male	235	55.6
Female	188	44.4
		
*Stage*		
T1	22	5.2
T2	76	17.9
T3	249	58.9
T4	64	15.2
Not available	12	2.8
		
*Nodal*		
N0	214	50.6
N1	100	23.6
N2	95	22.5
Not available	14	3.3
		
*Metastasis*		
M0	368	87
M1	40	9.5
Not available	15	3.5
		
*Grade*		
G1	4	0.9
G2	303	71.6
G3	109	25.8
Not available	7	1.7
		
*R status*		
R0	377	89.1
R1	22	5.2
R2	2	0.5
Not available	22	5.2
		
*Localization*		
Appendix	1	0.2
Coecum	47	11.1
Ascendens	91	21.5
Flexura dextra	7	1.7
Transversum	29	6.9
Flexura sinistra	7	1.7
Descendens	18	4.3
Sigma	76	18
Rectum	101	23.9
Rectosigmoid	23	5.4
Multifocal	3	0.7
Not available	20	4.6
		
*KRAS status*		
WT	327	77.3
MUT	53	12.5
Not available	43	10.2
		
*BRAF status*		
WT	53	12.5
MUT	5	1.2
Not available	365	86.3
		
*PI3K status*		
WT	15	3.6
MUT	0	0
Not available	408	96.4
		
*MSI status*		
MSI-H	2	0.5
MSI-L	3	0.7
MSS	4	0.9
Not available	414	97.9

Abbreviation: MSI: microsatellite instability.
