# Supplementary figures and images for: Uncoupling of EGFR–RAS signaling and nuclear localization of YBX1 in colorectal cancer
Source: Oncogenesis. 2016 Jan 18;5(1):e187–. doi: 10.1038/oncsis.2015.51 (PMC4728680; doi:10.1038/oncsis.2015.51)

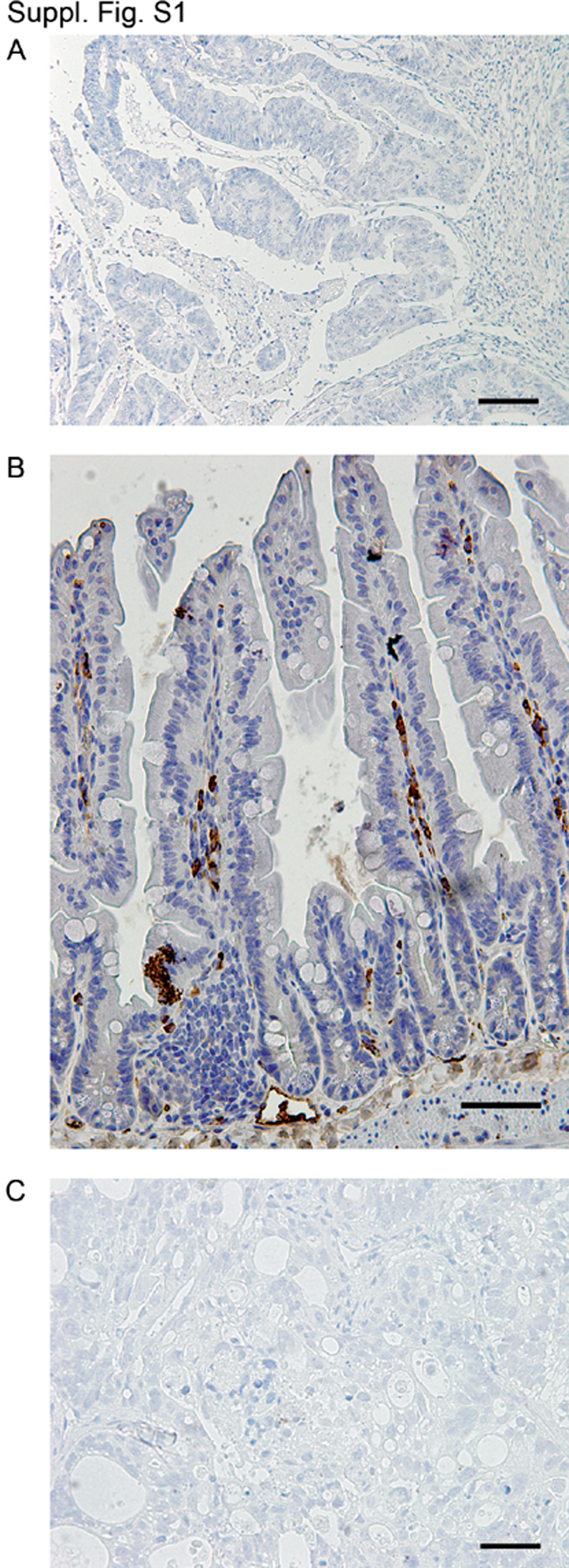

Supplement: Supplementary Figure 1 [file oncsis201551x1.tif]

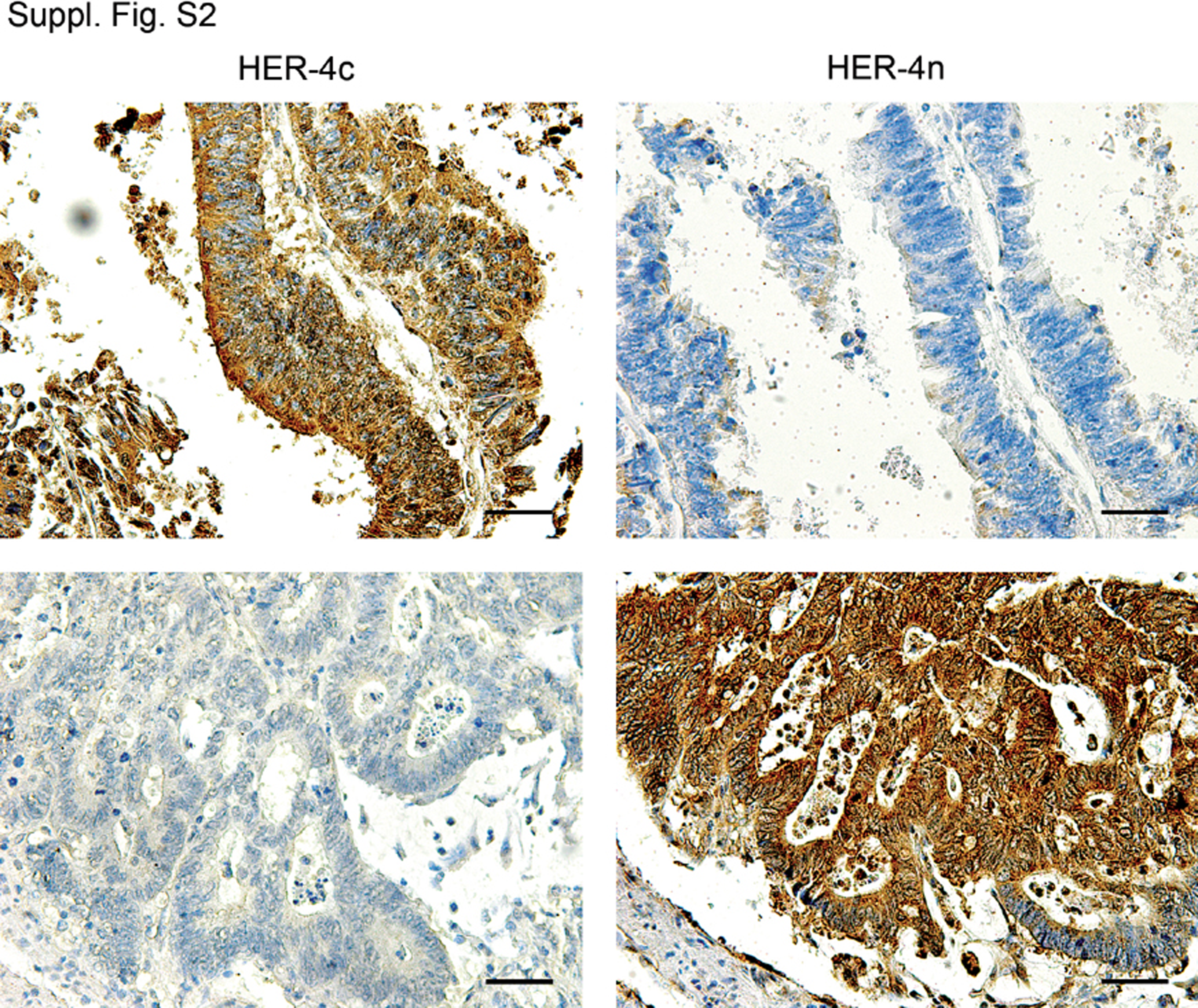

Supplement: Supplementary Figure 2 [file oncsis201551x2.tif]

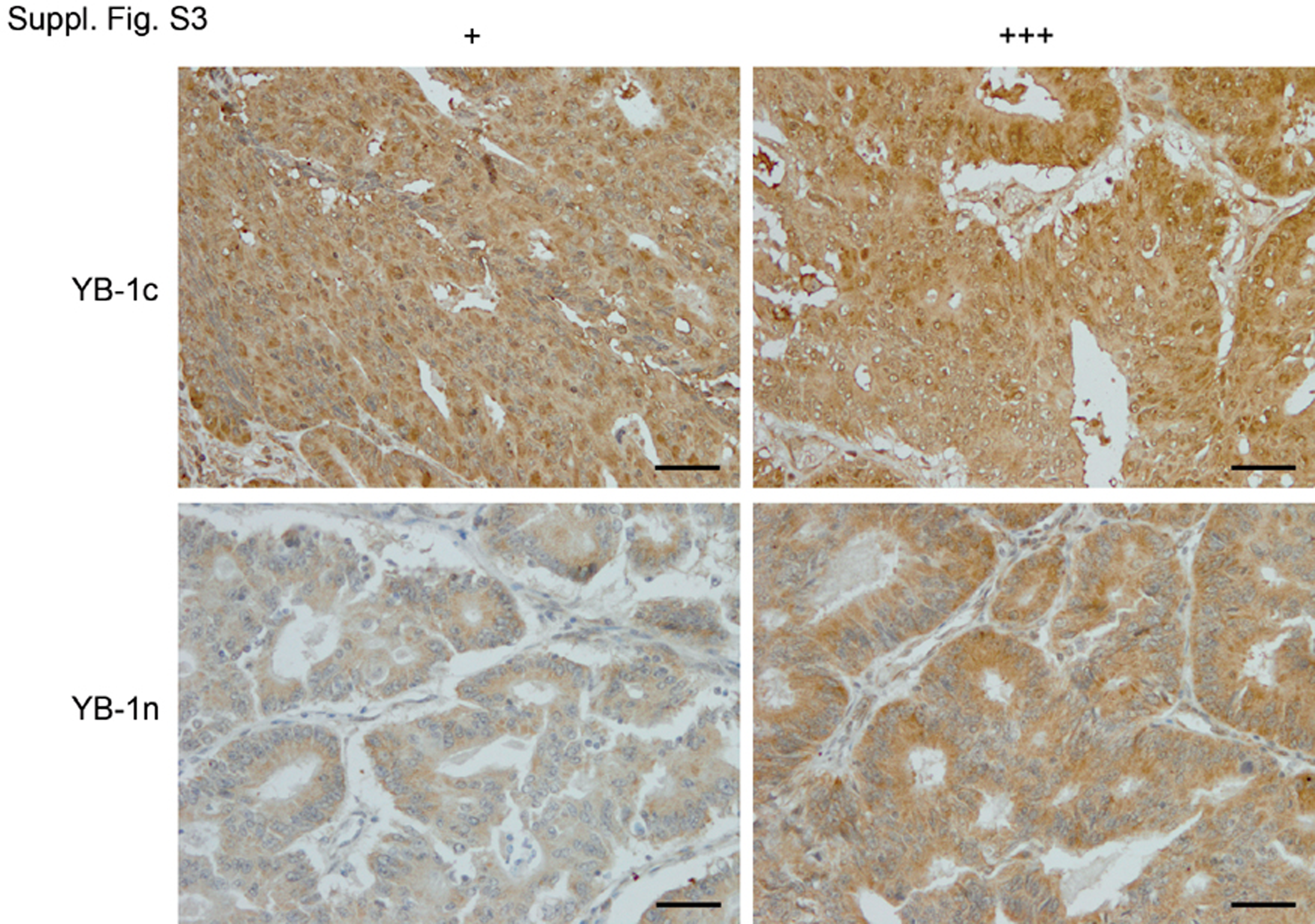

Supplement: Supplementary Figure 3 [file oncsis201551x3.tif]

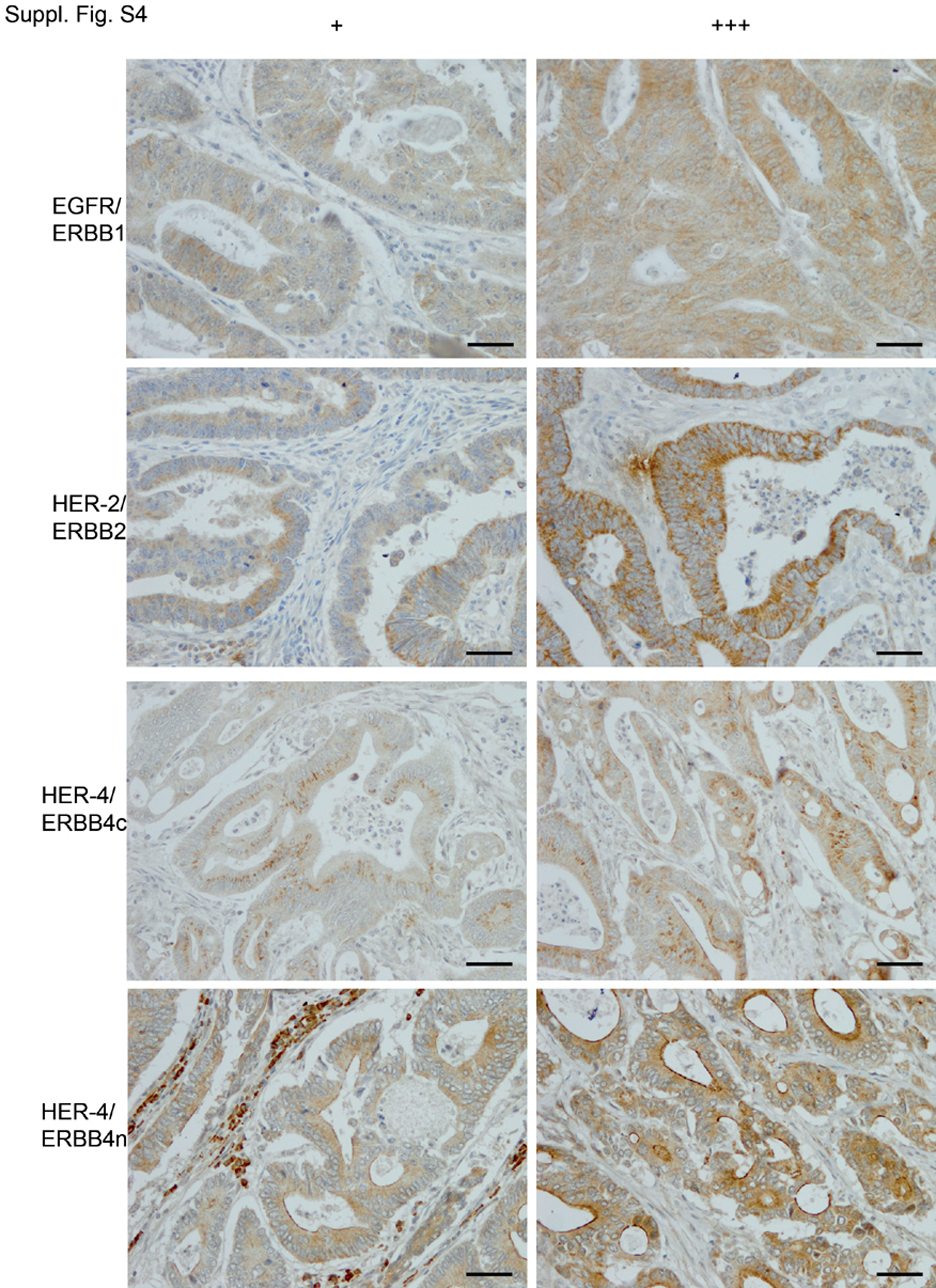

Supplement: Supplementary Figure 4 [file oncsis201551x4.tif]

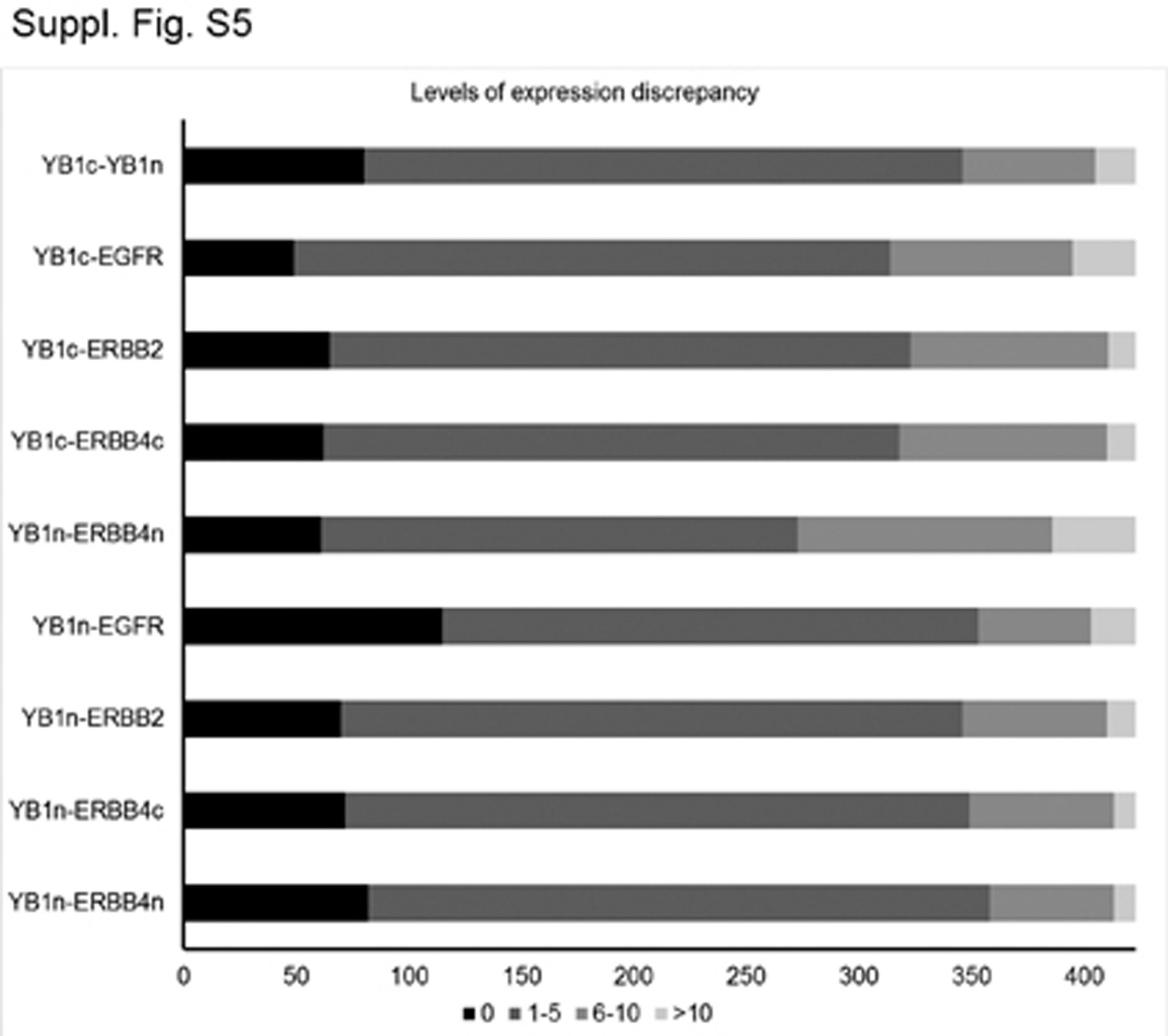

Supplement: Supplementary Figure 5 [file oncsis201551x5.tif]

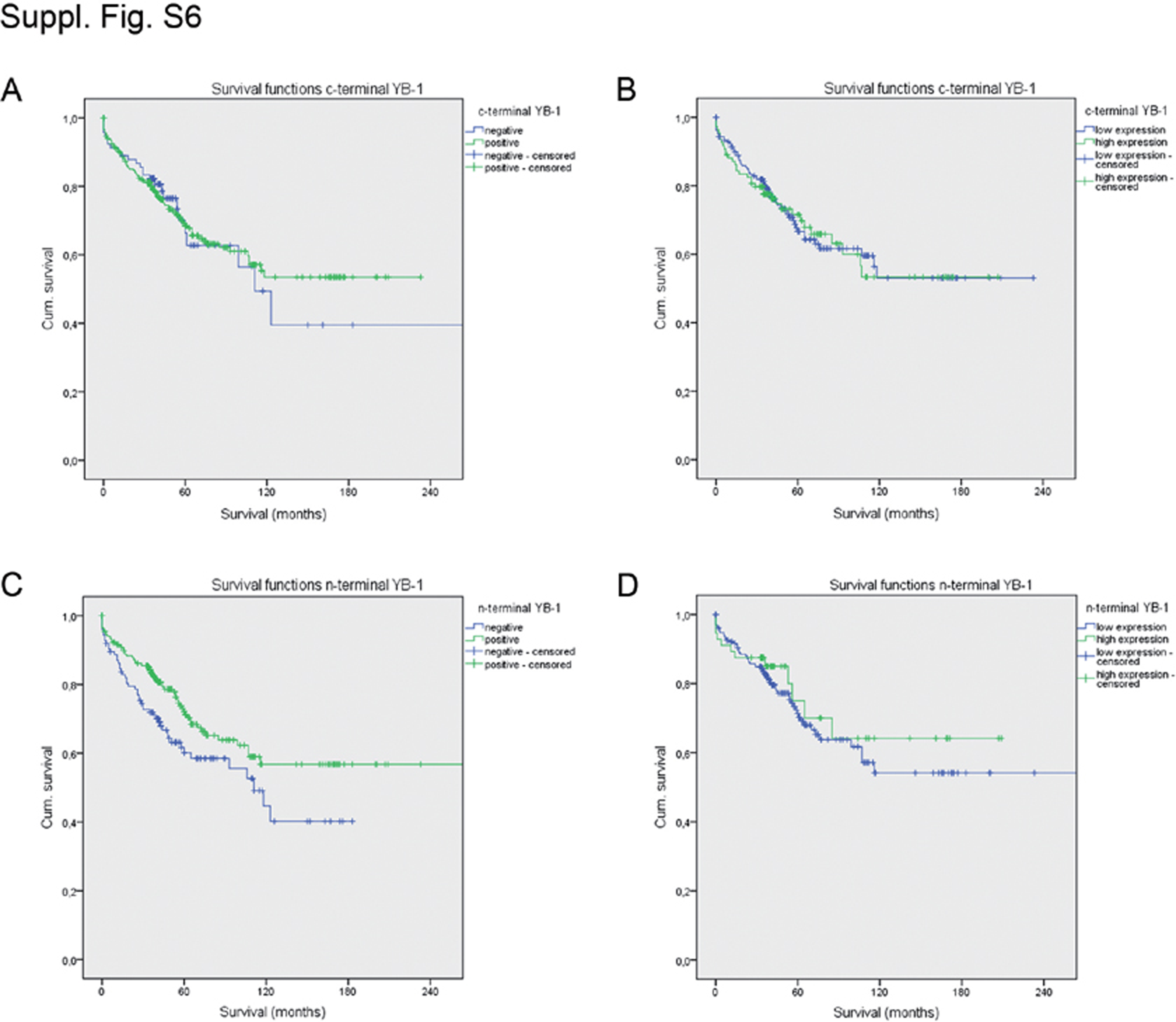

Supplement: Supplementary Figure 6 [file oncsis201551x6.tif]

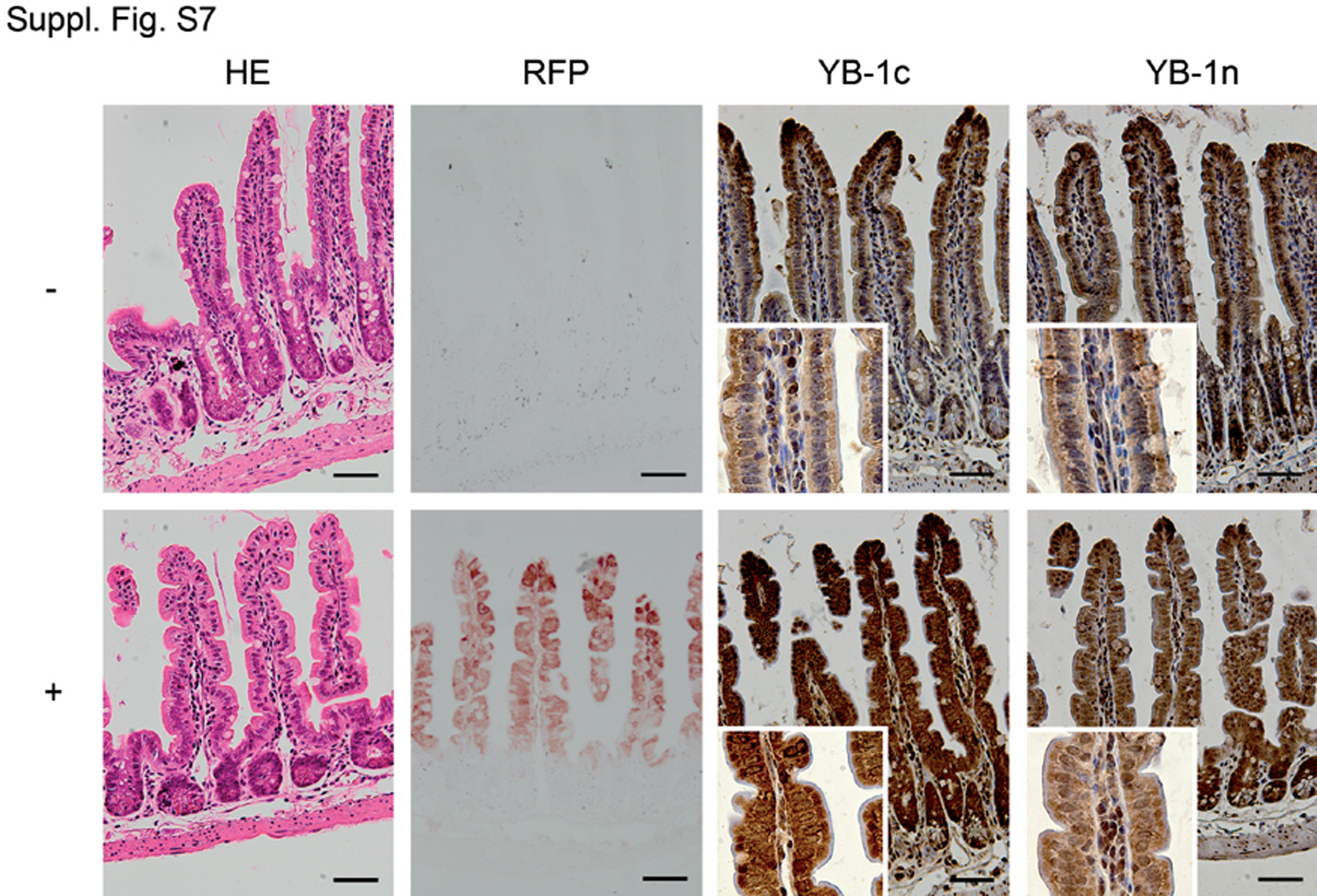

Supplement: Supplementary Figure 7 [file oncsis201551x7.tif]

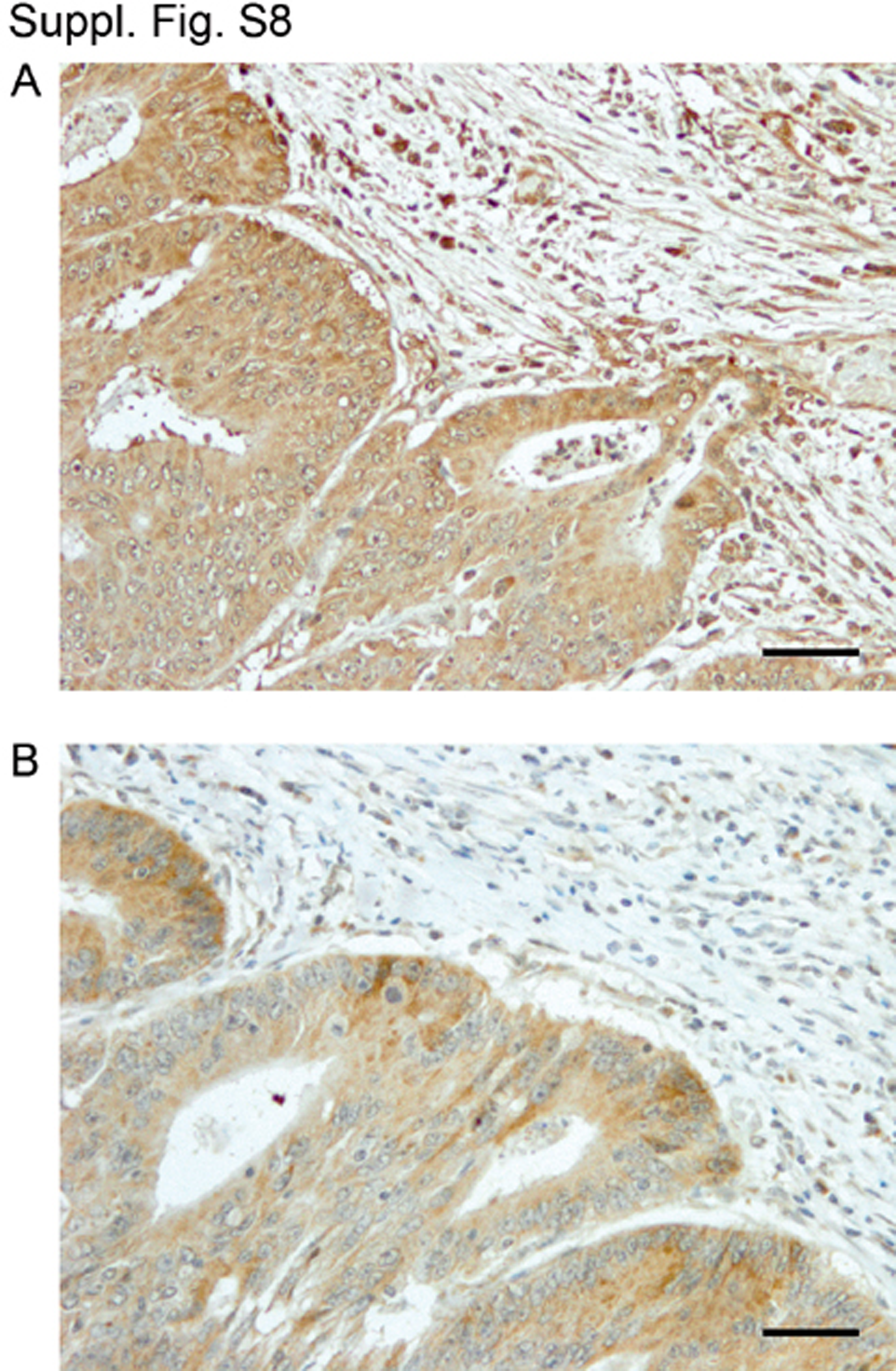

Supplement: Supplementary Figure 8 [file oncsis201551x8.tif]
