# Supplementary material for: Uncoupling of EGFR–RAS signaling and nuclear localization of YBX1 in colorectal cancer
Source: Oncogenesis. 2016 Jan 18;5(1):e187–. doi: 10.1038/oncsis.2015.51 (PMC4728680; doi:10.1038/oncsis.2015.51)
Supplement: Supplementary Figure Legends [file oncsis201551x9.doc]

**Figure S1**

Immunohistochemistry negative controls without primary antibodies. **(A)** Human tissue micro arrays. **(B)** Mouse intestine with negative mucosal layer and aberrant focal staining of mucin, endothelial and some inflammatory cells in the lamina propria. Scale bar: 50 µm. **(C)** CaCo2 colorectal cell line xenograft. Scale bar: 100 µm.

**Figure S2**

Immunohistochemical staining of c-terminal HER4/ERBB4 and n-terminal HER4/ERBB4 showing the same tumor sample with marked differences in staining. Scale bar: 100 µm.

**Figure S3**

Immunohistochemical staining of YB-1 in full section tissue samples with faint staining differences in different tumor areas. Scale bar: 100 µm.

**Figure S4**

Immunohistochemical staining of EGFR, ERBB2 and ERBB4 antibodies in full section tissue samples with faint staining differences in different tumor areas. Scale bar: 100 µm.

**Figure S5**

Levels of expression discrepancy between antibodies. X-axis: number of samples. Y-axis: name of antibodies.

**Figure S6**

(A) Kaplan-Meier plot for survival analysis of YBX1c positive (+) and YBX1c negative (-) expression (p=0,977). (B) Kaplan-Meier plot for survival analysis for low YBX1c and high YBX1c expression (p=0,922).

(C) Kaplan-Meier plot for survival analysis of YBX1n positive (+) and YBX1n negative (-) expression (p=0,016). (D) Kaplan-Meier plot for survival analysis of low YBX1n and high YBX1n expression (p=0,450).

**Figure S7**

Immunohistochemical staining of YBX1n and YBX1c in the intestine from mice harboring a doxycycline inducible *KRAS^G12V^* transgene, four days following KRAS induction in vivo (**+** lower panel) and in control samples without KRAS induction (**-** upper panel). HE: Hematoxylin–eosin-stained paraffin sections, RFP: immunhistochemical staining of red fluorescence protein indicating transgene expression, YBX1c and YBX1n. Scale bar: 50 µm. Insets: highlights of cytoplasmic and nuclear staining.

**Figure S8**

Immunohistochemical staining of (A) c-terminal YB-1 and (B) n-terminal YB-1 in full section tissue samples, showing no significant change in expression at the invasion front. Scale bar: 100 µm.
